# Identification of a novel broadly HIV-1-neutralizing antibody from a CRF01_AE-infected Chinese donor

**DOI:** 10.1038/s41426-018-0175-1

**Published:** 2018-11-01

**Authors:** Bin Ju, Dan Li, Li Ren, Jiali Hou, Yanling Hao, Hua Liang, Shuo Wang, Jiang Zhu, Min Wei, Yiming Shao

**Affiliations:** 1 School of Medicine, Nankai University, 300071 Tianjin, China; 2 Nankai University Second People’s Hospital, School of Medicine, Nankai University, 300071 Tianjin, China; 3 State Key Laboratory of Infectious Disease Prevention and Control, National Center for AIDS/STD Control and Prevention, Chinese Center for Disease Control and Prevention, Collaborative Innovation Center for Diagnosis and Treatment of Infectious Diseases, 102206 Beijing, China; 4 Department of Immunology and Microbial Science, The Scripps Research Institute, La Jolla, CA 92037 USA

## Abstract

The isolation and characterization of monoclonal broadly neutralizing antibodies (nAbs) from natural HIV-1-infected individuals play very important roles in understanding nAb responses to HIV-1 infection and designing vaccines and therapeutics. Many broadly nAbs have been isolated from individuals infected with HIV-1 clade A, B, C, etc., but, as an important recombinant virus, the identification of broadly nAbs in CRF01_AE-infected individuals remains elusive. In this study, we used antigen-specific single B-cell sorting and monoclonal antibody expression to isolate monoclonal antibodies from a CRF01_AE-infected Chinese donor (GX2016EU04), a broad neutralizer based on neutralizing activity against a cross-clade virus panel. We identified a series of HIV-1 monoclonal cross-reactive nAbs, termed F2, H6, BF8, F4, F8, BE7, and F6. F6 could neutralize 21 of 37 tested HIV-1 Env-pseudotyped viruses (57%) with a geometric mean value of 12.15 μg/ml. Heavy and light chains of F6 were derived from IGHV4-34 and IGKV 2-28 germlines, complementarity determining region (CDR) 3 loops were composed of 18 and 9 amino acids, and somatic hypermutations (SHMs) were 16.14% and 11.83% divergent from their respective germline genes. F6 was a GP120-specific nAb and recognized the linear epitope. We identified for the first time a novel broadly HIV-1-neutralizing antibody, termed F6, from a CRF01_AE-infected donor, which could enrich the research of HIV-1 nAbs and provide useful insights for designing vaccine immunogens and antibody-based therapeutics.

## Introduction

Acquired immunodeficiency syndrome (AIDS), mainly caused by the infection of human immunodeficiency virus type-1 (HIV-1), increases the risk of opportunistic infections and malignancy and leads to a high morbidity and mortality. As a type of retrovirus, HIV-1 contains M, O, and N groups and displays a high degree of genetic diversity. HIV-1 M group is classified into nine divergent subtypes (A, B, C, D, F, G, H, J, and K) and multiple recombinant forms including circulating recombinant forms (CRFs) (CRF01_AE, CRF07_BC, etc.) and unique recombinant forms (URFs) (AC, AD, etc.). Envelope (Env) amino acid sequences can differ by ~20% within a particular subtype and 35% between different subtypes^1–3^. The predominant HIV-1 strains in China include clade B, CRF01_AE, and CRF07_BC/CRF08_BC^4,5^.

After several years of natural infection, 10–25% of HIV-1 infected individuals may develop cross-reactive nAbs, some of which could neutralize the majority of viruses from multiple genetic subtypes worldwide^6–9^. Identifying these nAbs and describing their characteristics can provide important information for the design of HIV-1 vaccines and immunotherapies. Currently, a variety of monoclonal nAbs including VRC-CH31, PG9, PG16, PGT121 (all from clade A-infected donors)^10–12^, VRC01, 10E8, 35O22, N123-VRC34.01, A16, DRVIA7 (all from clade B-infected donors)^5,9,13–16^, PGT135 (clade C)^12^, VRC-PG04 (AD recombinant)^10^, Y498 (CRF07_BC)^17^, and PGT125 (CRF02_AG)^12^ have been isolated from different individuals.

HIV-1 CRF01_AE, originating from central Africa and spreading epidemically in Asia, is the first large-scale epidemic of a recombinant strain in the world and was first identified among female sex workers in northern Thailand in 1989^18–22^. CRF01_AE is responsible for 5% of cases in the world and plays an important role in regional epidemics, the majority of which are found in South Asia, Southeast Asia, and East Asia^23,24^. CRF01_AE was first identified in China among persons in the southwest provinces of Yunnan and Guangxi in the early 1990s and has emerged as a widespread strain in nationwide HIV-1 infections^19,25–27^. Despite extensive attempts to isolate broadly nAbs, as an important recombinant virus, the analysis of monoclonal nAbs in CRF01_AE-infected donors remains unsuccessful.

In the current study, we measured and evaluated the breadth and potency of neutralizing antibody responses in a cohort of CRF01_AE-infected Chinese subjects using a multi-subtype panel of viruses. The data demonstrated that some top neutralizing subjects could neutralize over 90% of tested Env-pseudotyped viruses (not published), which provided us an opportunity to isolate broadly nAbs from CRF01_AE-infected donors. In this study, we focused on the identification of monoclonal nAbs from a top neutralizing subject GX2016EU04 and isolated a novel broadly nAb termed F6 with a neutralizing breadth of ~57% against 37 tested HIV-1 isolates.

## Results

### Isolation of antigen-specific single B cells

**Fig. 1:**
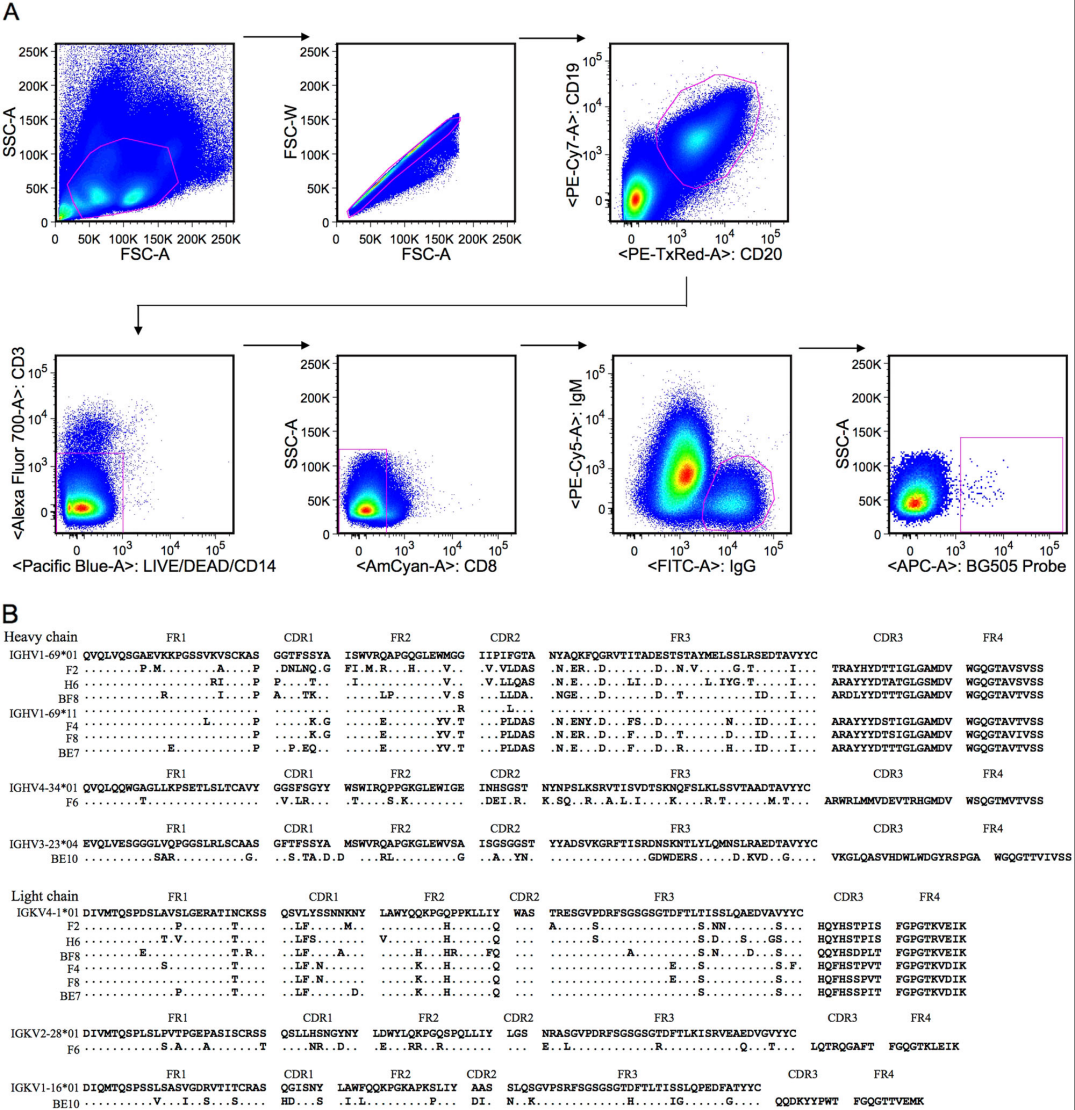
Identification of eight monoclonal antibodies from a HIV-1 CRF01_AE-infected Chinese donor. **a** Isolation of antigen-specific single B cells by flow cytometry. Single cells were sorted into a 96-well PCR plate containing lysis buffer according to the presented gating strategy. **b** Amino acid sequence analysis of monoclonal antibodies with alignment to respective germline genes. The framework region (FR) and complementarity determining region (CDR) were determined based on the program IMGT/V-QUEST. The symbol “.” denotes conserved amino acids

Antigen-specific single B cells were isolated from PBMCs of a HIV-1 CRF01_AE-infected Chinese donor by flow cytometry using the sorting probe BG505. As shown in Fig. 1a, CD19 + CD20 + CD3-CD14-CD8-IgG + IgM-BG505 + B cells were gated and sorted into a 96-well PCR plate. Single-cell PCR and sequencing were used to amplify and analyze variable region genes of monoclonal antibodies. Eight matching heavy and light chain antibody genes were acquired and cloned into the full IgG1 expression vectors. The amino acid sequences of monoclonal antibodies aligned to the relevant germline genes are shown in Fig. 1b.

### Genetic analysis of monoclonal antibodies

The program IMGT/V-QUEST (www.imgt.org/IMGT_vquest/vquest) was used to analyze variable genes of monoclonal antibodies. As shown in Table 1, heavy chains of F2, H6, BF8, F4, F8, and BE7 were derived from the germline IGHV1-69, CDR3 loops were composed of 17 amino acids, and SHMs were 14.24–18.06% divergent. Light chains of F2, H6, BF8, F4, F8, and BE7 belonged to the germline IGKV4-1, CDR3 loops contained 9 amino acids, and SHMs were 7.80–11.70% divergent. The heavy and light chains of F6 were derived from IGHV4-34 and IGKV 2-28, CDR3 loops were composed of 18 and 9 amino acids, and SHMs were 16.14% and 11.83% divergent. The heavy and light chains of BE10 belonged to IGHV3-23 and IGKV1-16, CDR3 loops contained 21 and 9 amino acids, and SHMs were 19.44% and 10.61% divergent.

**Table 1:** Gene family analysis of monoclonal antibodies

	Heavy chain					Light chain			
	IGHV	IGHJ	IGHD	CDR3 (aa)	SHM (%)	IGKV	IGKJ	CDR3 (aa)	SHM (%)
F2	1–69*01	3*01	3–22*01	17	18.06	4–1*01	3*01	9	9.93
H6	1–69*01	3*01	3–22*01	17	17.01	4–1*01	3*01	9	11.70
BF8	1–69*01	3*01	3–22*01	17	14.24	4–1*01	3*01	9	10.64
F4	1–69*11	3*01	3–22*01	17	16.32	4–1*01	3*01	9	8.87
F8	1–69*11	3*01	3–22*01	17	14.58	4–1*01	3*01	9	7.80
BE7	1–69*11	3*01	3–22*01	17	16.32	4–1*01	3*01	9	7.80
F6	4–34*01	6*01	2–8*01	18	16.14	2–28*01	2*02	9	11.83
BE10	3–23*04	6*02	3–16*02	21	19.44	1–16*01	1*01	9	10.61

The program IMGT/V-QUEST was applied to analyze the gene germline, complementarity determining region (CDR) 3 length, and somatic hypermutation (SHM). The SHM frequency was calculated from the mutated nucleotides

### The expression and characterization of monoclonal binding antibodies

**Fig. 2:**
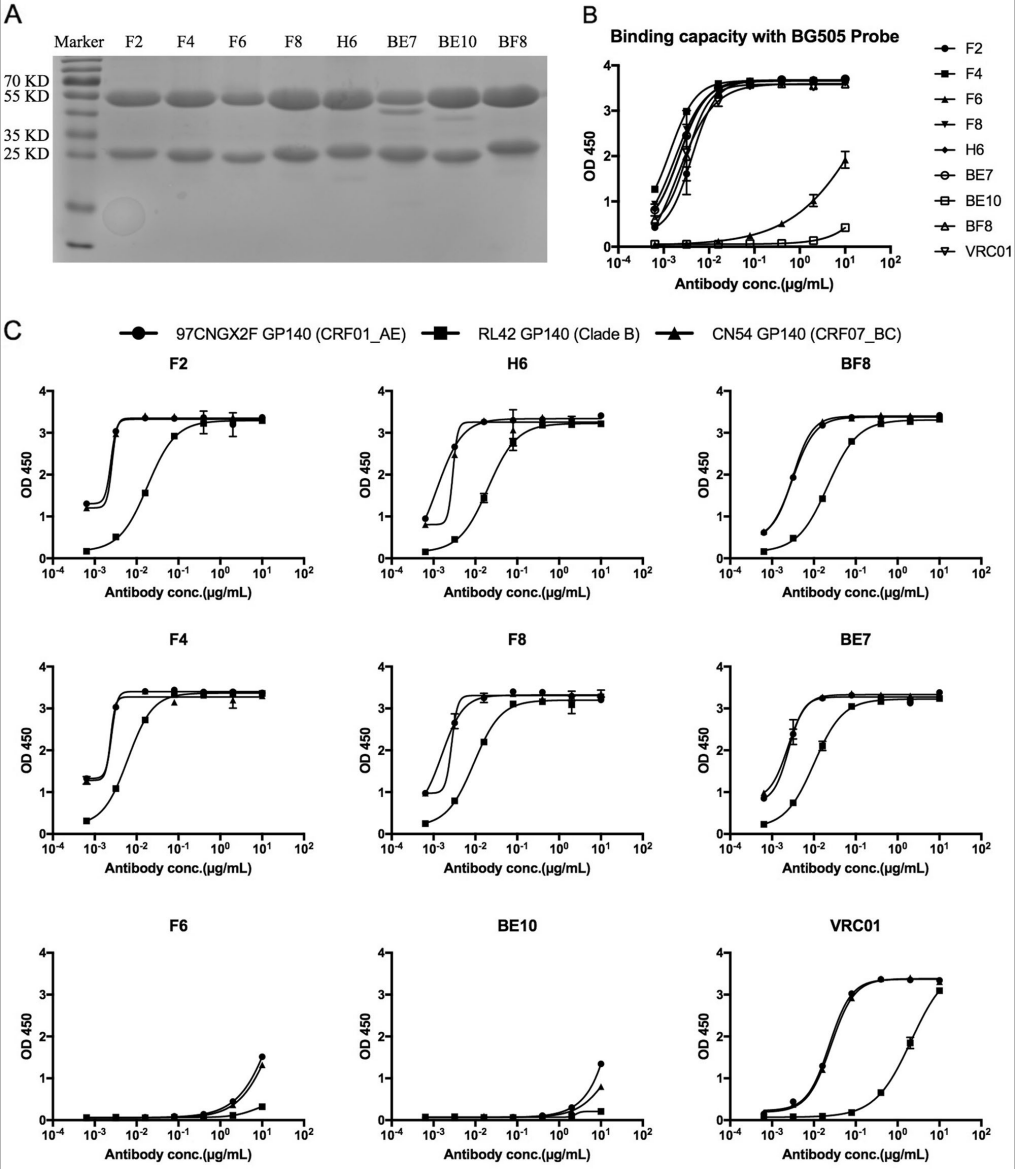
Purification and identification of monoclonal binding antibodies. **a** Reducing 12% SDS-PAGE analysis of monoclonal antibodies. **b** ELISA binding of monoclonal antibodies with the sorting probe BG505. **c** ELISA of cross-reactive monoclonal antibodies binding with diverse HIV-1 GP140 antigens including clade B, CRF01_AE, and CRF07_BC

The monoclonal antibodies were expressed and purified with 293 F cells and protein A columns. The yield of F2 was 2.12 mg/L culture supernatant, and the rest of the monoclonal antibodies also had good expression quantities (F4: 25.30 mg/L, F6: 3.83 mg/L, F8: 28.51 mg/L, H6: 17.00 mg/L, BE7: 13.04 mg/L, BE10: 28.06 mg/L, and BF8: 25.85 mg/L). A reducing SDS-PAGE analysis is shown in Fig. 2a, with the heavy chain proteins found at a position of ~55 KD and the protein bands of light chains at ~25 KD. To confirm the specificity of these antibodies for the HIV-1 antigen, we detected ELISA binding of each monoclonal antibody to sorting probe BG505 (clade A). As shown in Fig. 2b, F2, H6, BF8, F4, F8, and BE7 displayed stronger binding capacities than F6 and BE10. ELISA was also used to detect the cross-reactivity of each monoclonal antibody binding to cross-subtype HIV-1 antigens. As shown in Fig. 2c, F2, H6, BF8, F4, F8, and BE7 bound more strongly than F6 and BE10 to GP140 antigens of RL42 (clade B), 97CNGX2F (CRF01_AE), and CN54 (CRF07_BC).

### The neutralizing activity of monoclonal antibodies

A TZM-bl/pseudovirus assay was used to test the neutralizing activity of each monoclonal antibody. As shown in Table 2A, F2, H6, BF8, F4, F8, and BE7 were all cross-reactive nAbs that could neutralize viruses SF162 and MW965. Meanwhile, BF8 neutralized virus 398F1, F4 neutralized viruses 398F1 and 25710, and BE7 neutralized virus 25710. F6 could neutralize 8 of 12 Tier 2 viruses on the Global Panel, which seemed to qualify it as a broadly nAb. Unfortunately, BE10 could not neutralize the 14 tested viruses. Then, we used the DRVI Panel to further detect the neutralizing activity of F6. As shown in Table 2B, F6 could neutralize 13 of 25 cross-clade viruses, including two Tier 3 viruses (TRJO4551 and CH120). Taken together, F6 neutralized 21 of 37 tested HIV-1 isolates. The neutralizing breadth of F6 was ~57%, and the geometric mean potency of neutralized viruses was 12.15 μg/ml.

**Table 2A:** Neutralizing activity of monoclonal antibodies on the Global Panel and two Tier 1 viruses from the DRVI Panel

				Global panel											
	Pseudovirus	SF162	MW965	398F1	TRO11	X2278	25710	CE0217	CE1176	X1632	CNE55	CNE8	BJOX2000	CH119	246F3
	Clade	B	C	A	B	B	C	C	C	G	CRF01_AE	CRF01_AE	CRF07_BC	CRF07_BC	AC
	Tier	1	1	2	2	2	2	2	2	2	2	2	2	2	2
F2		**0.06**	*0.38*	>50	>50	>50	>50	>50	>50	>50	>50	>50	>50	>50	>50
H6		*0.34*	*0.99*	>50	>50	>50	>50	>50	>50	>50	>50	>50	>50	>50	>50
BF8		**0.04**	**0.12**	23.98	>50	>50	>50	>50	>50	>50	>50	>50	>50	>50	>50
F4		**0.04**	**0.02**	49.42	>50	>50	38.26	>50	>50	>50	>50	>50	>50	>50	>50
F8		**0.01**	**0.18**	>50	>50	>50	>50	>50	>50	>50	>50	>50	>50	>50	>50
BE7		**0.02**	**0.04**	>50	>50	>50	44.19	>50	>50	>50	>50	>50	>50	>50	>50
F6		>50	>50	10.00	>50	31.94	46.00	>50	13.53	8.21	41.07	34.85	>50	>50	8.27
BE10		>50	>50	>50	>50	>50	>50	>50	>50	>50	>50	>50	>50	>50	>50
VRC01		*0.48*	**0.07**	*0.41*	*0.77*	*0.35*	*0.87*	*0.42*	4.88	*0.31*	*0.64*	*0.56*	>50	*1.56*	*0.51*

Neutralizing potency was measured as IC_50_ in μg/ml of the monoclonal antibodies. Values <0.2 μg/ml in bold, 0.2–2 μg/ml in italic, and 2–50 μg/ml in underline

**Table 2B:** Neutralizing activity of F6 against diverse HIV-1 isolates on the DRVI Panel

	Pseudovirus	Q461	Q769	Q259	Q842	SF162	QH0692	SC422661	PVO.4	AC10	RHPA4259	REJO4541	TRJO4551	CAAN5342
	Clade	A	A	A	A	B	B	B	B	B	B	B	B	B
	Tier	2	2	2	2	1	2	2	3	2	2	2	3	2
F6		>50	3.89	>50	>50	>50	21.78	25.57	>50	17.29	19.28	>50	2.58	31.78
VRC01		*0.65*	**0.12**	**0.18**	**0.09**	*0.48*	*1.72*	*0.51*	*1.09*	3.51	**0.12**	*0.34*	*0.41*	*1.72*
	Pseudovirus	MW965	ZM109	Du422	ZM249M	CAP45	CH110	CH117	CH181	CH120	BJMSM2249	BJMSM2316	BJMSM2498	
	Clade	C	C	C	C	C	CRF07_BC	CRF07_BC	CRF07_BC	CRF07_BC	CRF01_AE	CRF01_AE	CRF01_AE	
	Tier	1	1	2	2	2	2	2	2	3	ND	ND	ND	
F6		>50	>50	18.97	>50	>50	17.82	>50	>50	43.56	2.50	*0.91*	*1.56*	
VRC01		**0.07**	*0.52*	>50	*0.24*	9.09	*1.34*	*0.36*	*1.14*	6.90	3.77	*0.32*	*1.50*	

*ND* not determined

Neutralizing potency was measured as IC_50_ in μg/ml of the monoclonal antibodies. Values <0.2 μg/ml in bold, 0.2–2 μg/ml in italic, and 2–50 μg/ml in underline

### The newly isolated F6 recognizes the linear epitope on GP120

**Fig. 3:**
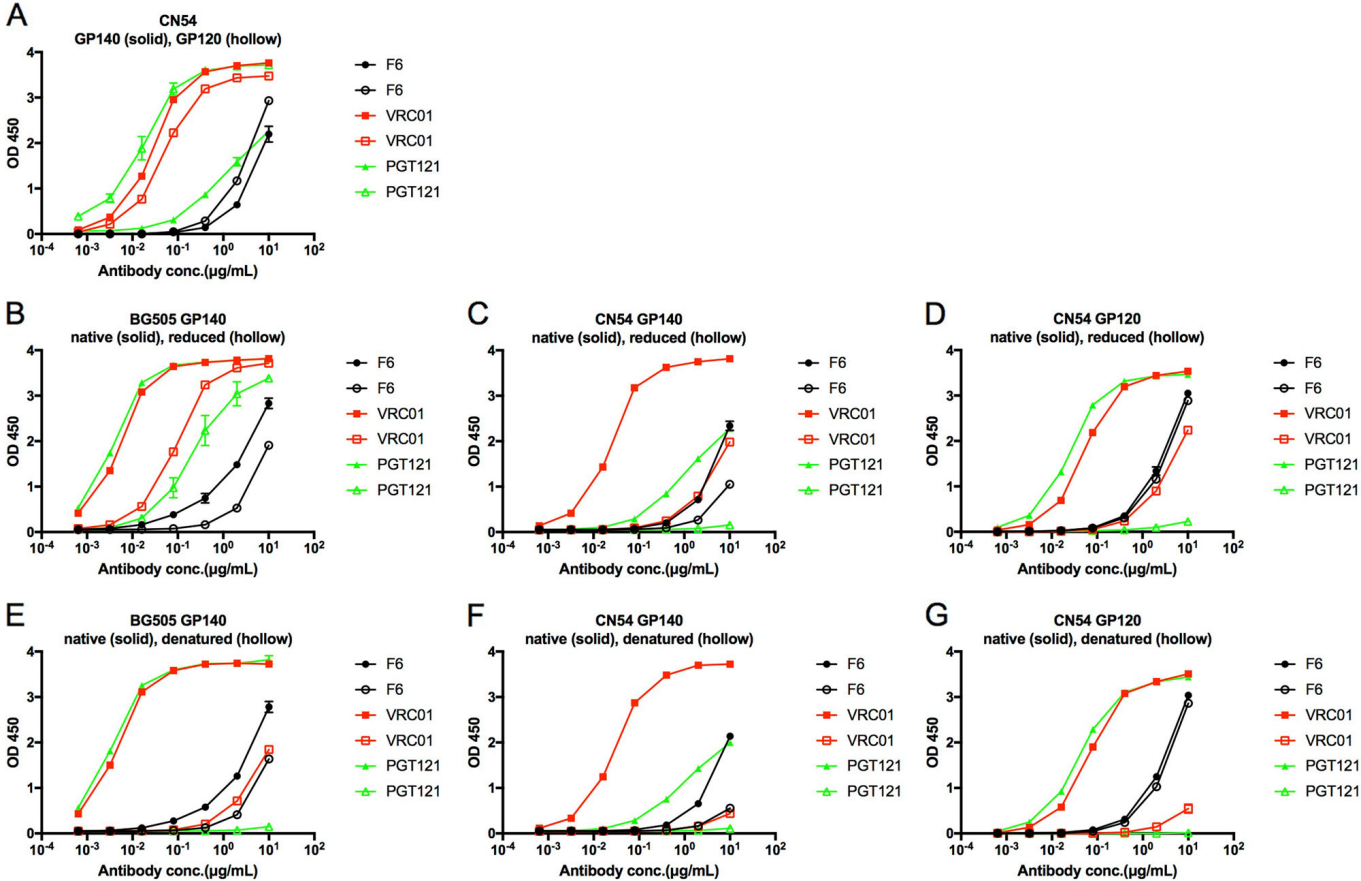
The newly isolated F6 recognizes the linear epitope on GP120. **a** ELISA binding of F6 to HIV-1 GP140 and GP120 antigens. **b**–**d** ELISA binding of F6 to native and reduced BG505 GP140, CN54 GP140, and CN54 GP120 treated with 10 mM DTT at 37 °C for 1 h. **e**–**g** ELISA binding of F6 to native and denatured BG505 GP140, CN54 GP140, and CN54 GP120 treated with denaturing buffer (New England Biolabs) at 100 °C for 10 min

To explore the recognizing epitope of F6, we first tested its binding capacity to HIV-1 GP120. As shown in Fig. 3a, F6 and control nAbs (VRC01 and PGT121) could bind to CN54 GP140 and GP120 proteins. Unlike some conformational epitope-specific nAbs (VRC01 and PGT121), the binding activity of F6 to reduced BG505 and CN54 GP140 was slightly decreased compared to native proteins (Fig. 3b, c), and DTT treatment could not influence the binding of F6 to CN54 GP120 (Fig. 3d). A similar result was found in denatured GP140 and GP120 proteins, which could strongly unfold the conformational epitope (Fig. 3e–g).

### F6 does not target the CD4 binding site epitope of GP120

**Fig. 4:**
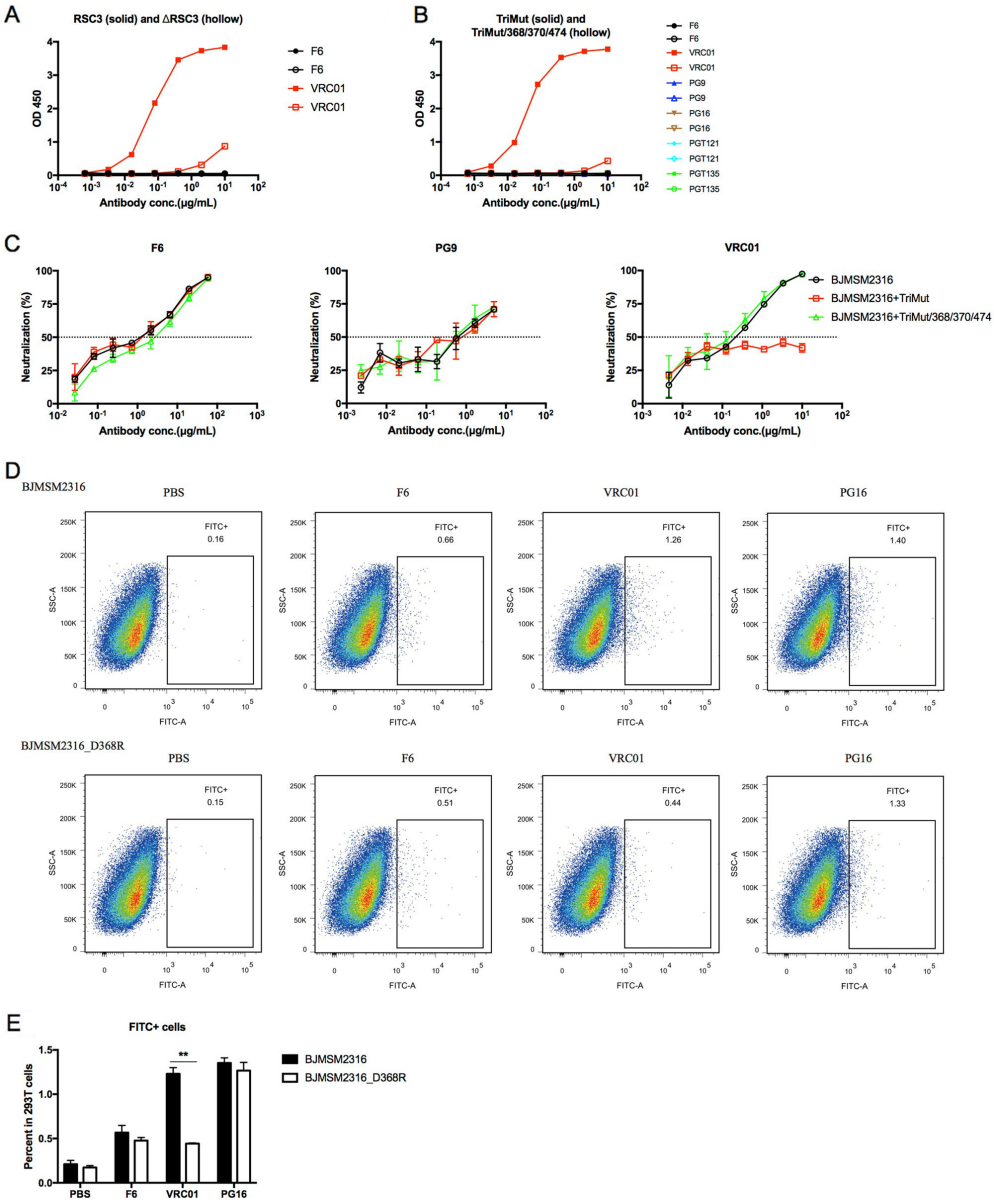
F6 does not target the CD4 binding site epitope of GP120. **a** ELISA binding of F6 to RSC3 and ΔRSC3. **b** ELISA binding of F6 to TriMut and TriMut/368/370/474. **c** Competition neutralizing of F6 against HIV-1_BJMSM2316_ with TriMut and TriMut/368/370/474. **d** Flow cytometry dot plots showing the binding capacity of F6 to HIV-1_BJMSM2316_ and HIV-1_BJMSM2316_D368R_ GP160 on the surface of transfected 293 T cells. **e** The percentage of FITC + cells in 293 T cells are shown as mean ± SD (*n* = 3). The symbol “**” indicates *P* < 0.01

As a major group of anti-HIV-1 nAbs, we tried to determine whether F6 belongs to the class of CD4 binding site-directed antibodies or not. Unlike the CD4bs-directed antibody VRC01, F6 could not bind RSC3 and ΔRSC3 (Fig. 4a). A similar result was found in another pair of CD4bs-specific proteins. VRC01 bound TriMut strongly and bound TriMut/368/370/474 weakly. F6 and other non-CD4bs nAbs (PG9, PG16, PGT121, and PGT135) could not bind these two proteins (Fig. 4b). The neutralization of VRC01 against HIV-1_BJMSM2316_ was inhibited by TriMut but not by TriMut/368/370/474. F6 and PG9 could still neutralize HIV-1_BJMSM2316_ in the presence of TriMut or TriMut/368/370/474 (Fig. 4c). The CD4bs epitope of HIV-1_BJMSM2316_ was disrupted by a D to R mutation at position 368. The binding capacity of VRC01 to HIV-1_BJMSM2316_D368R_ GP160 declined significantly, but F6 and PG16 were not influenced (Fig. 4d, e).

### Alternative epitope identification of F6

**Fig. 5:**
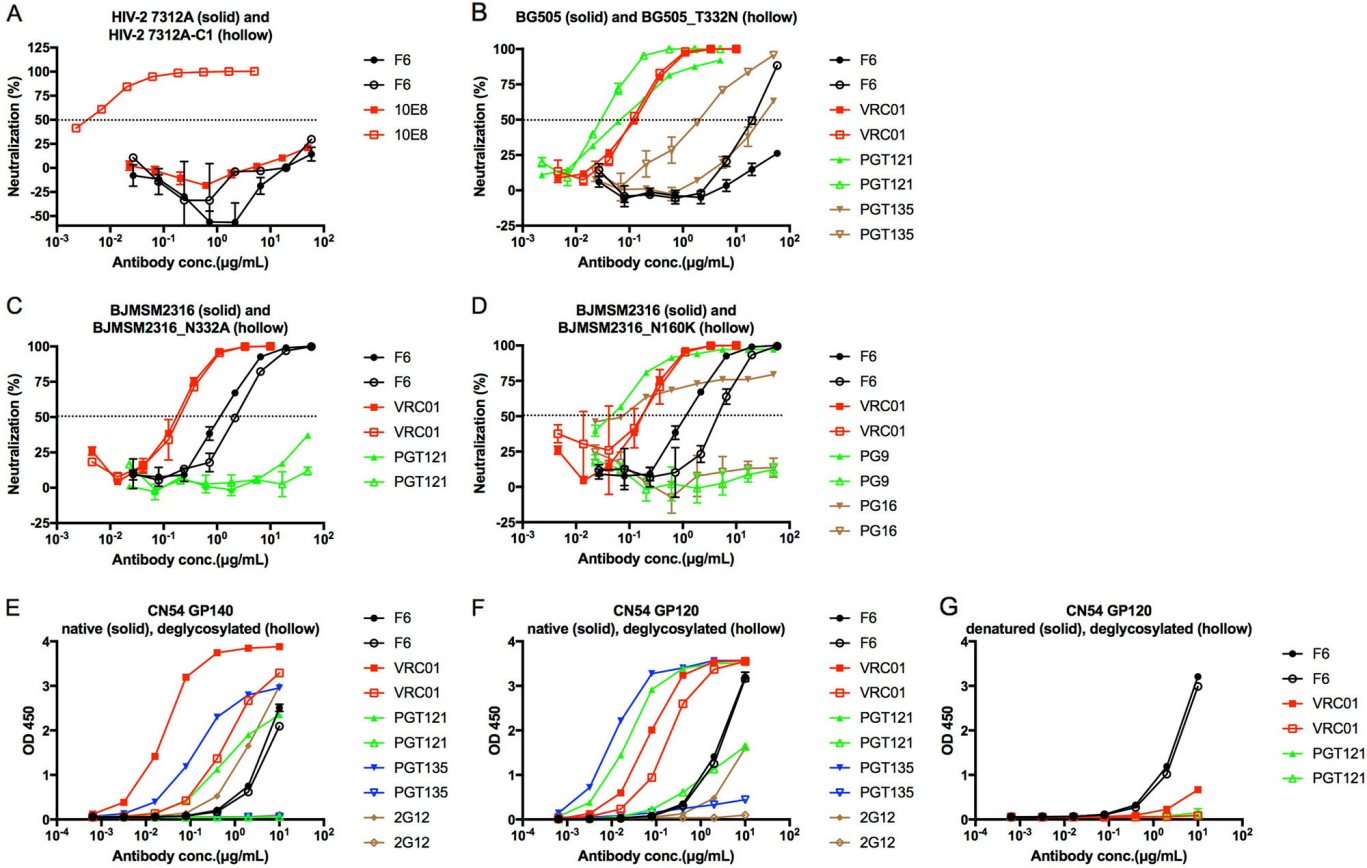
Alternative epitope identification of F6. **a** Neutralization of F6 against HIV-2_7312A_ and HIV-2_7312A-C1_. **b** Neutralization of F6 against HIV-1_BG505_ and HIV-1_BG505_T332N_. **c** Neutralization of F6 against HIV-1_BJMSM2316_ and HIV-1_BJMSM2316_N332A_. **d** Neutralization of F6 against HIV-1_BJMSM2316_ and HIV-1_BJMSM2316_N160K_. **e**–**g** ELISA binding of F6 to deglycosylated CN54 GP140 and GP120 treated with Endo H enzyme (New England Biolabs) at 37 °C according to the manufacturer’s protocol

A membrane proximal external region (MPER)-directed antibody 10E8 could neutralize HIV-2_7312A-C1_ but could not neutralize HIV-2_7312A_. F6 could not neutralize HIV-2_7312A-C1_ and HIV-2_7312A_ (Fig. 5a). HIV-1_BG505_ contains an N to T polymorphism (nonpermissive for N-glycosylation) at position 332, and a mutation of T to N at this residue could reverse its escape from F6 neutralization (Fig. 5b). We then constructed an N332A site-directed mutant based on HIV-1_BJMSM2316_, and the neutralization of F6 against HIV-1_BJMSM2316_N332A_ was slightly decreased (Fig. 5c). A similar result was found in another pair of mutants (HIV-1_BJMSM2316_ and HIV-1_BJMSM2316_N160K_). The mutation of N to K at position 160 of HIV-1_BJMSM2316_ resulted in a limited decrease in its neutralizing sensitivity to F6 (Fig. 5d). Deglycosylation of CN54 GP140 or GP120 strongly decreased its binding activity with some glycan-directed nAbs (PGT121, PGT135, and 2G12), but F6 and VRC01 were not influenced (Fig. 5e–g).

## Discussion

In our previous studies, Hu and colleagues evaluated the prevalence, breadth, and potency of nAb responses in HIV-1 clade B and CRF07_BC infected Chinese individuals. A total of 29% of plasma samples neutralized more than 80% of the tested viral strains, and 64% neutralized more than 50% in Chinese individuals infected with HIV-1 clade B^28^. The proportions of neutralizing breadths >80 and 50% were 18 and 53% in CRF07_BC-infected Chinese individuals, respectively^29^. Our group and colleagues identified a monoclonal nAb (DRVIA7) with modest neutralizing breadth from a HIV-1 B-infected Chinese donor, which was the first VRC01-like antibody isolated from Chinese donors and provided an opportunity to investigate the crucial events in the early stages of VRC01-like antibody development. VRC01-like neutralizing heavy-chain precursors rapidly matured within 2 years, and a less favorable light-chain interaction with gp120 glycans resulted in limited neutralizing activity and stalled lineage development^16^. The Dr. He laboratory at the Chinese Academy of Medical Sciences also attempted to isolate monoclonal nAbs from Chinese HIV-1-infected individuals. Two CD4 binding site-directed antibodies, A16 (32%) and Y498 (30%), were respectively identified from an HIV-1 B-infected and a CRF07_BC-infected Chinese donor by panning a phage display Fab library. These two monoclonal antibodies neutralized HIV-1 isolates with limited breadth, similar to that of b12 (41%) and HJ16 (36%)^5,17^. In a current study, we also measured and evaluated the nAb responses in a cohort of CRF01_AE-infected Chinese individuals, suggesting that 22% of plasma samples neutralized more than 80% of tested viruses and that 71% neutralized more than 50% (not published). Overall, the percentage of broad neutralizers in CRF01_AE-infected individuals was not the lowest among these three cohorts, but broadly nAbs had not been isolated from CRF01_AE-infected donors. In the present study, we focused on the isolation and characterization of anti-HIV-1 monoclonal nAbs from a CRF01_AE-infected Chinese donor whose serum exhibited broadly neutralizing activity, resulting in the identification of a series of HIV-1 binding and neutralizing antibodies by antigen-specific single B cell sorting and monoclonal antibody expression.

F2, H6, BF8, F4, F8, and BE7 belonged to the same antibody class according to their gene analysis, binding capacity, and neutralizing activity. They were derived from the same germline gene with the same CDR3 length and a similar gene sequence. These cross-reactive binding antibodies strongly bound to HIV-1 antigens of clade A, B, CRF01_AE, and CRF07_BC, and might be used to develop antigen detection and purification reagents. They were all extremely potent nAbs against two Tier 1 viruses of clade B and clade C. Meanwhile, BF8, F4, and BE7 could neutralize one or two Tier 2 viruses of clade A and/or clade C with modest potency. In previous clinical trials, though some immunogens could elicit high titers of nAbs against Tier 1 viruses, neutralization of Tier 2 viruses was difficult to induce^30–32^. Similarly, Sanders and colleagues used a soluble recombinant HIV-1 envelope glycoprotein trimer to induce nAbs in rabbits and macaques, which demonstrated that cross-reactive nAbs against more sensitive (Tier 1 and autologous Tier 2) viruses could be induced, however, heterologous Tier 2 viruses were difficult to neutralize^33^. Taken together, though F2, H6, BF8, F4, F8, and BE7 exhibited high binding affinity and neutralizing potency against Tier 1 viruses, they could not neutralize Tier 2 viruses broadly. Neutralizing antibody responses do not correlate directly with binding antibody responses, and inducing a Tier 2 virus response will not be achieved simply by increasing the titer of Tier 1 nAbs, as entirely different antibody specificities are likely involved.

As immunotherapeutic agents, monoclonal broadly nAbs are important tools for the prevention, control, and eradication of HIV-1. Moldt and colleagues reported that passive transfer of PGT121 efficiently protected against challenge of simian-human immunodeficiency virus (SHIV) in macaques^34^. The mechanism of PGT121-mediated protection against SHIV was demonstrated by Liu and colleagues, which involved the clearance of infectious virus in distal tissues^35^. Meanwhile, other preclinical studies demonstrated that passive administration of PGT126 protected against both vaginal and rectal SHIV challenges, and engineered tri-specific antibody VRC01/PGDM1400-10E8v4 conferred complete immunity against a mixture of SHIVs in nonhuman primates (NHPs)^36,37^. Bolton and colleagues^38^ showed that nAbs suppressed acute SHIV plasma viremia and limited seeding of cell-associated viral reservoirs in macaques. In two treatments of chronic infection, nAb administration suppressed plasma viral loads and resulted in significantly reduced proviral DNA levels in PBMCs and lymph nodes in chronically SHIV-infected macaques, and a single infusion of VRC01 induced a 1.1 to 1.8 log10 reduction in plasma viremia in chronically HIV-1-infected humans^39,40^.

F6 is the first broadly HIV-1-neutralizing antibody identified from a CRF01_AE-infected donor. In this study, F6 neutralized 21 viruses of clades A, B, C, G, CRF07_BC, CRF01_AE, and AC on a panel of 37 Env-pseudotyped viruses; 57% was a modest level of neutralizing breadth compared with other current broadly nAbs, such as CD4bs-directed antibodies: A16 (32%), b12 (41%), VRC01 (91%), VRC-PG04 (76%), VRC-CH31 (83%), and N6 (98%);^5,9,10,41^ V1V2 glycan site-directed antibodies: PG9 (79%) and PG16 (73%);^11^ V3 glycan supersite-directed antibodies: 2G12 (32%), PGT121 (70%), PGT125 (52%), and PGT135 (33%);^12^ MPER-directed antibodies: 4E10 (96%), 2F5 (57%), and 10E8 (98%);^12,13^ and GP41-GP120 interface-directed antibodies: 35O22 (62%), PGT151 (66%), and N123-VRC34.01 (49%)^14,15,42,43^. With the development of isolating and genetic engineering technologies, a number of nAbs have been identified from natural infection subjects and large-scale clinical trials have already begun to evaluate the safety and efficacy of nAbs in human volunteers^44,45^. F6, as an HIV-1 broadly nAb, could be used in future research of passive protection and therapy. According to a preliminary epitope analysis, F6 was a GP120-specific nAb and recognized the linear epitope. F6 did not belong to the class of CD4bs or MPER-directed nAbs. Though a mutation of T to N at the 332 residue of HIV-1_BG505_ reversed its escape from F6 neutralization and the neutralizing sensitivity of HIV-1_BJMSM2316_ to F6 was declined, to a certain extent the substitution of N332A or N160K, resulting in the removal of glycans from envelope proteins by the Endo H enzyme, did not influence its binding activity with F6. Some N-linked glycosylation sites on the HIV-1 envelope seemed to have some impact on the recognition of F6, but the existing data indicated that F6 might not simply recognize glycans to neutralize viruses. Though we narrowed the possible epitope of F6, we did not identify its exact recognition epitope in this study, which hopefully can be elucidated by mapping epitopes in more detail and determining the crystal structure of F6 in a future study. The structural characterization of nAb-Env interactions could provide substantial insights into the mechanism of HIV-1 neutralization and help in the design of immunogens to induce nAbs^46,47^. Jardine and colleagues demonstrated that an engineered germline-targeting GP120 outer domain immunogen (eOD-GT6) could activate both germline and mature VRC01-class B cells and should be used as a prime vaccine candidate^48^. Medina-Ramirez and colleagues reengineered a BG505 SOSIP.v4.1-GT1 trimer, which could bind multiple broadly nAb germline precursors in vitro and activate B cells in immunization experiments^49^.

In conclusion, we first isolated HIV-1 nAbs from a CRF01_AE-infected donor, filling this knowledge gap of nAb responses in subjects infected with diverse HIV-1 subtypes. We provided a series of HIV-1 binding and neutralizing antibodies, which could serve as antigen detection reagents and candidate immunotherapeutic agents used in passive protection and treatment against HIV-1 infection and could serve as vaccine templates to guide structure-based immunogen design by exploring their recognizing and neutralizing mechanisms in the future.

## Materials and methods

### Ethics and human subjects

This study was reviewed and approved by the ethics committee of the Chinese Center for Disease Control and Prevention and performed in accordance with relevant guidelines and regulations. Peripheral blood mononuclear cells (PBMCs) collected from subject GX2016EU04 were leftovers from a previous study. GX2016EU04 was infected with a HIV-1 CRF01_AE virus and had not initiated antiretroviral treatment at the time of PBMCs sampling. Serum neutralizing breadth was over 90% against diverse clades of HIV-1 Env-pseudotyped viruses (not published).

### Isolation of antigen-specific single B cells by flow cytometry

Thawed PBMCs were stained with an antibody cocktail consisting of CD19-PE-Cy7, CD3-Alexa Fluor 700, CD14-Pacific Blue, CD8-BV510, IgM-PE-Cy5, IgG-FITC (all from BD Biosciences), CD20-ECD (Beckman), and BG505 Probe-APC (kindly provided by The Scripps Research Institute). The sorting probe BG505 was a biotin-labeled HIV-1_BG505_ GP140 trimer^50^ conjugated with streptavidin-allophycocyanin (SA-APC) (Invitrogen). A LIVE/DEAD Fixable Dead Cell Stain Kit (Pacific Blue) (Invitrogen) was used to exclude dead cells. Antigen-specific single B cells were gated as CD19+CD20+CD3-CD14-CD8-IgM-IgG+BG505+ and sorted into a 96-well PCR plate containing lysis buffer. The sorted plate was snap-frozen on dry ice and stored at −80 °C^9^. Flow cytometric data were acquired on an Aria SORP flow cytometer (BD Biosciences) and analyzed with the software FlowJo (TreeStar).

### Single B cell PCR, gene family analysis, and expression of monoclonal antibodies

The IgG heavy and light chain gene products were amplified by single B cell PCR and cloned into eukaryotic expression vectors to produce full IgG1 antibodies as previously described^10,16,51^. Briefly, the frozen plate was thawed at room temperature. RT-PCR and nested PCR were performed to amplify variable regions of monoclonal antibodies, and the positive PCR products were purified and sequenced. The IgG heavy and light chain nucleotide sequences of the variable region were analyzed by the program IMGT/V-QUEST (www.imgt.org/IMGT_vquest/vquest)^5,17^. Matching antibody variable genes were separately cloned into full-length IgG1 heavy and light chain expression vectors containing constant region sequences (kindly provided by the Vaccine Research Center)^52^. Monoclonal antibodies were expressed by cotransfection of 293 F cells (Life Technologies) with equal amounts of paired heavy and light chain plasmids and purified from culture supernatants using protein A columns (National Engineering Research Center for Biotechnology, Beijing) according to the manufacturer’s protocol.

### Enzyme linked immunosorbent assay

The binding capacity of monoclonal antibodies was measured by enzyme linked immunosorbent assay (ELISA). Briefly, purified 2 μg/ml HIV-1 antigens (expressed in our lab and provided by The Scripps Research Institute) were coated into 96-well plates at 4 °C overnight. The plates were washed five times with PBST (0.05% Tween 20 in PBS) and blocked with blocking buffer (5% skim milk and 2% bovine albumin in PBS) at RT for 1 h. Monoclonal antibodies were serially 5-fold diluted from 10 μg/ml in blocking buffer, and then added into wells and incubated at 37 °C for 1 h. Plates were washed five times with PBST, and HRP conjugated goat anti-human IgG antibodies (ZSGB-BIO, Beijing) were added at 1:5000 diluted in blocking buffer and incubated at 37 °C for 1 h. Finally, TMB substrate (Kinghawk, Being) was added and incubated for 20 mins at RT, and the reaction was stopped by the addition of 2 M H_2_SO_4_ to each well. The readout was measured at a wave length of 450 nm.

### Neutralizing assay

The neutralizing activity of monoclonal antibodies was detected by TZM-bl/pseudovirus assay as previously described^53^. Briefly, monoclonal antibodies were serially 3-fold diluted with DMEM and incubated with 200 TCID_50_ pseudovirus at 37 °C for 1 h. TZM-bl cells (1 × 10^4^/well) containing 11 μg/ml DEAE-dextran were added into the plate and incubated at 37 °C for 48 h. The cells were lysed, and the luciferase activity was measured by Bright-Glo Luciferase (Promega, USA) according to the manufacturer’s protocol. The 50% inhibitory concentration (IC_50_) was calculated as the antibody concentrations required to inhibit infection by 50%. A Global Panel of reference Env clones was established by the Montefiori group at Duke University, which facilitated highly standardized assessments of neutralizing antibodies across multiple HIV-1 research platforms in different parts of the world^54^. Another separate panel containing 25 viruses was compiled by the Shao group at the Division of Research on Virology and Immunology (DRVI Panel) of China CDC, which reflected the HIV-1 epidemic in China and has been utilized in previous studies to evaluate neutralizing antibody responses^16,28,29^. Pseudoviruses HIV-1_BG505_T332N_, HIV-1_BJMSM2316_N160K_, and HIV-1_BJMSM2316_N332A_ were generated by site-directed mutagenesis based on HIV-1_BG505_ and HIV-1_BJMSM2316_ in this study.

### Competition neutralizing assay

To map the neutralization epitope of the newly isolated antibody F6, a competition neutralizing assay was performed as previously described^55^. Briefly, TZM-bl cells (1 × 10^4^/well) were plated in DMEM the day before. F6 was serially 3-fold diluted and incubated with competitive inhibitors, TriMut or TriMut/368/370/474 (paired CD4 binding site-specific proteins, kindly provided by The Scripps Research Institute), at 37 °C for 1 h. Pseudoviruses were added into the antibody/inhibitor mixtures and incubated at 37 °C for 1 h. Then, the above antibody/inhibitor/pseudovirus mixtures were added into TZM-bl cells and incubated at 37 °C for 48 h. The cells were lysed and a luciferase activity was measured as described in a direct neutralizing assay. Three neutralization curves (medium, TriMut, and TriMut/368/370/474) were obtained and used to determine whether or not F6 recognizes the CD4 binding site epitope.

### Flow cytometry analysis

Briefly, 293 T cells were respectively transfected with HIV-1_BJMSM2316_ and HIV-1_BJMSM2316_D368R_ GP160 expressing plasmids and harvested after 48 h. Cells were incubated with F6 (10 μg/ml) or other control nAbs (VRC01 and PG16) at 37 °C for 30 min and then detected by 1:1000 diluted goat anti-human IgG-FITC (Invitrogen). Flow cytometric data were acquired on an LSR Fortessa flow cytometer (BD Biosciences) and analyzed with FlowJo software (TreeStar). Statistical analyses were performed with a *t* test using the software GraphPad Prism 5.01. *P* < 0.05 was considered significant.
